# Supplementary material for: The interactome of intact mitochondria by cross-linking mass spectrometry provides evidence for coexisting respiratory supercomplexes
Source: Mol Cell Proteomics. 2017 Dec 8;17(2):216–32. doi: 10.1074/mcp.RA117.000470 (PMC5795388; doi:10.1074/mcp.RA117.000470)
Supplement: Supplemental Data [file supp_RA117.000470_133922_0_supp_23978_fzffwf.zip › spectra_annotation/mito_spectra_annotation/227-1-3-1-13-1.pdf]

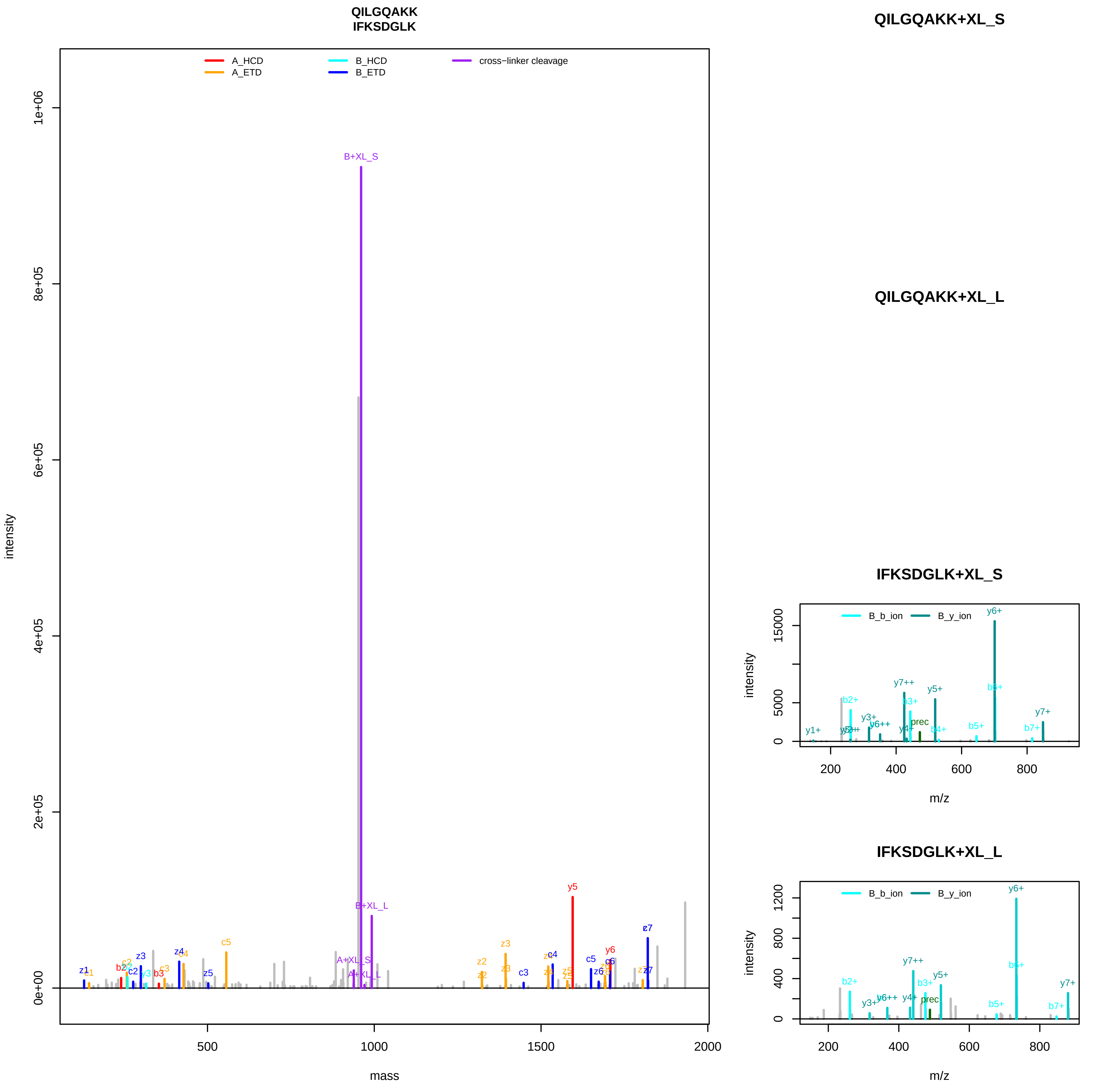

QILGQAKK
IFKSDGLK
A_HCD
A_ETD
B_HCD
B_ETD
cross−linker cleavage
B+XL_S
B+XL_L
A+XL_S
A+XL_L
y5
y6
c5
z3
z4
c4
z7
c7
z1
z2
z5
z6
c2
c3
c6
b2
b3
y3
intensity
mass
0e+00
2e+05
4e+05
6e+05
8e+05
1e+06
500
1000
1500
2000
QILGQAKK+XL_S
QILGQAKK+XL_L
IFKSDGLK+XL_S
B_b_ion
B_y_ion
y6+
y7++
y5+
b6+
b2+
b3+
y3+
y6++
prec
y4+
b4+
b5+
b7+
y7+
y1+
intensity
m/z
0
5000
15000
200
400
600
800
IFKSDGLK+XL_L
B_b_ion
B_y_ion
y6+
y7++
y5+
b6+
b2+
b3+
y3+
y6++
y4+
prec
b5+
b7+
y7+
intensity
m/z
0
400
800
1200
200
400
600
800
